# Author Correction: Genomes of fungi and relatives reveal delayed loss of ancestral gene families and evolution of key fungal traits

**DOI:** 10.1038/s41559-025-02881-7

**Published:** 2025-10-01

**Authors:** Zsolt Merényi, Krisztina Krizsán, Neha Sahu, Xiao-Bin Liu, Balázs Bálint, Jason E. Stajich, Joseph W. Spatafora, László G. Nagy

**Affiliations:** 1 Synthetic and Systems Biology Unit, Institute of Biochemistry, Biological Research Centre, Szeged, Hungary; 2 Department of Botany and Plant Pathology, Oregon State University, Corvallis, OR USA; 3 Department of Plant Pathology and Microbiology, University of California, Riverside, CA USA; 4 Present Address: Institute of Forensic Genetics, Hungarian Institute for Forensic Sciences, Budapest, Hungary

**Keywords:** Molecular evolution, Phylogenetics, Genome evolution

Correction to: *Nature Ecology & Evolution* 10.1038/s41559-023-02095-9, published online 22 June 2023.

In the version of this article originally published, Extended Data Fig. 8 was an inadvertent duplication of Extended Data Fig. 9 and has now been updated in the HTML and PDF versions of the article.

